# Tuning of Essential Oil Properties by Enzymatic Treatment: Towards Sustainable Processes for the Generation of New Fragrance Ingredients

**DOI:** 10.3390/molecules19079203

**Published:** 2014-07-01

**Authors:** Sylvain Antoniotti

**Affiliations:** Institut de Chimie de Nice, UMR 7272 CNRS - Université Nice Sophia Antipolis, Parc Valrose, 06108 Nice, France; E-Mail: sylvain.antoniotti@unice.fr; Tel./Fax: +33-0-492-076-151

**Keywords:** biocatalysis, fragrance chemistry, green chemistry, natural extracts, remediation

## Abstract

In this review, several strategies of modification of essential oils by enzymatic treatment are presented. Being either applied before or after the production of the essential oil, enzymatic methods are shown to be particularly adapted to attain the required selectivity, specificity and efficiency in sustainable processes delivering products eligible for the natural grade. Examples dealing with the optimization of the properties of essential oils in terms of biological activity, odor and safety are provided, and it is likely that these strategies will address other type of properties in the future, such as the physico-chemical properties, for example.

## 1. Introduction

Essential oils are mixtures of highly hydrophobic organic compounds typically obtained from hydrodistillation of selected parts of a wide range of terrestrial plants, e.g. flowers, leafs, roots, and in the case of odoriferous woods, barks or heartwood.

Although they are mainly composed of terpenes, terpenoids and phenylpropanoids, they present a great diversity of chemical structures, chemical compositions, and as a consequence, of properties. Their most obvious property is their olfactory property, which has made essential oils the central ingredient of perfumery for centuries. If the odoriferous quality of essential oils is a commonly known biological property, it is not the only one. Some of their constituents could indeed present other types of biological activities either beneficial or deleterious for humans. As beneficial, one could note the use of lavender oil, rich in linalool and linalyl acetate, in the approved drug Silexan^®^, effective in the treatment of anxiety disorder [1]. Other beneficial effects such as antimicrobial and antifungal have been demonstrated for essential oils of lavender, thyme, rosemary, grapefruit, and many others sources and allowed their use as natural preservatives in cosmetic products [2,3,4] or in food [5]. One the dark side however, some constituents are confirmed or suspected toxic molecules, like methyleugenol [6], safrole [7], or hepatotoxic suspected agents such as estragole [8].

Since the composition of essential oils has a direct relationship with the qualitative and quantitative value of their properties, it could be useful to chemically modify the composition to increase a valuable property or attenuate an undesirable property. However, given the large number of compounds and functional groups found in essential oils, the selectivity towards a given target is hard to achieve, if not to say impossible, with conventional chemical procedures. For the removal of toxic compounds, strategies based on distillation are used at the industrial scale whenever possible, but they sometimes lack selectivity, induce uncontrolled rearrangements, and typically require energy.

In this context, our group has been interested lately in the use of enzymes to modify the composition of essential oils and tune their properties. Enzymatic processes seem indeed ideal to offer highly selective and substrate specific processes with a very good sustainability profile and low energy consumption [9,10]. Enzymes being involved in the metabolism of secondary metabolites, they could somehow be seen as the ideal reagent to modify natural products [11], and are being used to produce natural flavors and fragrances [12,13].

In this review, we wish to focus on the enzymatic modification of essential oils, and in particular those used in perfumery, featuring our recently published work in the field, some unpublished data and references from other groups. These enzymatic treatments could be performed either before or after the production of the essential oils. These two strategies have significantly different rationales, the latter consisting in an assisted extraction while the former is directed towards the chemical modification of some constituents. Out of the scope of this review is the enzymatic modification of vegetal oils for fatty acids incorporation [14,15] or the modification of functional properties of food proteins [16].

## 2. Discussion

Natural extracts are mixtures of organic molecules with similar physico-chemical properties but sometimes very divergent biological properties in a broad sense. For example, rose essential oil contains pleasant odorant molecules such as phenylethanol, citronellol, geraniol, β-damascone, β-damascenone and rose oxides, inert hydrocarbons such as 9-eicosene, nonadecane and others, non-terpenic hydrocarbons from the stearoptene, and the suspected human carcinogen methyleugenol [17]. The final properties of the natural extract, either desired or non-desired, are correlated with the concentrations of the relevant active compounds. Tuning these properties either with an enhancement or a reduction by enzymatic treatment could be envisaged, occurring either before or after the extraction process.

### 2.1. Pre-Treatment

**Figure 1:**
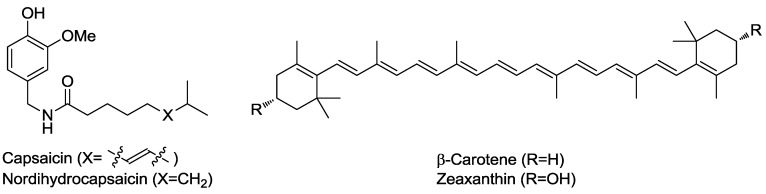
Capsaicinoids and carotenoids.

Enzymatic methods using cellulases have been used in the pre-treatment of plant materials to weaken the cells and facilitate their disruption to increase metabolites concentration in the extracting phase. The yield of steam distillation of spices, compared with the yield of hydrodistillation, could be enhanced by ca. 20% in the extraction of the volatile oil of cumin (*Cuminum cyminum L*.) by the use of hydrolytic enzymes such as cellulase, pectinase, protease and Viscozyme [18]. The chemical composition was examined, but no change could be observed comparing treated and untreated extracts confirming that no chemical transformation of the metabolites occured. Similarly, the extraction of capsaicinoids and carotenoids from chili guajillo “puya” (*Capsicum annuum L*.) flour after enzymatic treatment with enzyme preparations exhibiting pectolytic, cellulolytic, and carbohydrase activities allowed for increasing yields of the contents by up to 60% [19]. The methodology could be improved with the use of enzymatic extracts from *Rhizopus nigricans* allowing the recovery of 85% and 96% of the carotenoid and capsaicinoid contents, respectively (Figure 1) [20].

In the extraction of volatile oil of celery (*Apium graveolens* L.), the implementation of a cellulase, pectinase, protease or viscozyme pretreatment in the process resulted in ca. 25% improvement of the yield, while the sensory properties were unchanged [21].

The effect of a pre-treatment with a mixture of cellulase, β-glucanase, pectinase, and xylanase on the production of black pepper and cardamom extracts was investigated [22]. The enzymatic procedure not only allowed an increase of the yield in essential oil, but remarkably resulted in an increase of 70% of the β-caryophyllene content from black pepper and of 25% of the α-terpenyl acetate content from cardamom, these compounds being the major active components of the products. Similar results could be obtained with essential oils of *Thymus capitatus* and *Rosmarinus officinalis* leaves [23]. The essential oils were obtained in better yields after enzymatic pretreatment and exhibited higher concentration in carvacrol in the case of thyme and lower concentration in 1,8-cineole in the case of rosemary. Their antimicrobial activities against a series of pathogens (*Escherichia coli, Salmonella typhimurium, Streptococcus agalactiae, Staphylococcus aureus, Enterococcus feacium* and *Candida albicans*) were determined to be superior to essential oils obtained without pretreatment.

**Scheme 1:**
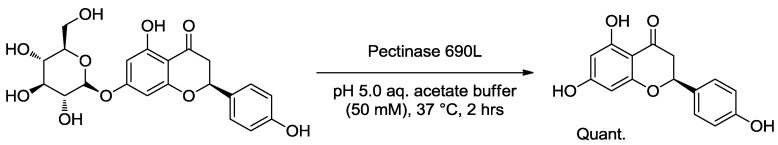
Deglycosylation of naringenin-7-O-glucoside from bergamot peel by pectinase.

Since some metabolites could exist in a glycosylated form, some enzymatic strategies have been developed to increase the final content in compounds of interest such as flavonoids from bergamot (*Citrus bergamia* Risso) peel using enzymes from *Aspergillus* sp. and *Trichoderma* sp. [24]. Up to 80%–90% of the flavonoids content were obtained after deglycosylation, due to β-glucosidase and α-rhamnosidase activities. This is an example where an enzymatic pretreatment is used to improve the biological properties of a natural extract based on the antioxidant activity of flavonoids (Scheme 1).

Similarly, the improvement of the olfactory quality of concretes and refined absolutes from *Osmanthus fragrans* Lour. flowers was realised by β-glucosidase-assisted hydrolysis [25]. Both improved yield and odor where observed with a significant increase of the amount of high-impact odorants nonanal, dihydro-β-ionol and (*E*)-β-ionone. In the vapor extraction of rose essential oil, the use of β-glucosidases allowed a modification of the chemical composition, and therefore of the olfactory properties [26]. Other examples on *Psoralea bituminosa* [27], *Syzygium aromaticum* (L.) merr. and perry (Myrtaceae) [28], and Hyssopus officinalis L. [29] were reported.

### 2.2. Post-Treatment

**Figure 2:**
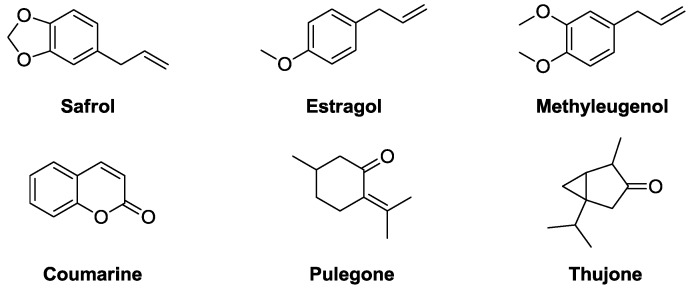
Chemicals from natural extracts in the spotlight for human toxicity.

A significant evolution of the manufacturing practices and the regulations occurred recently in the use of natural extracts in the cosmetic and perfume industry. In addition to the European Registration, Evaluation, Authorisation and Restriction of Chemical substances (REACH) regulation, a large number of chemicals found in natural extracts are under surveillance because of suspected or demonstrated toxicity such as carcinogen methyleugenol [6] and safrole [7], skin sensitisers such as atranol and derivatives from tree moss extract [30], or hepatotoxic estragole, for example (Figure 2) [8].

For these reasons, it is mandatory to keep their concentration low in final products, according to IFRA recommendations [31], and strategies to rectify their concentration in valuable extracts have become highly priced. Post-treatments by various distillation techniques are available, but require time-consuming procedure development, have high energy running costs, and often deliver product with altered quality, because of a lack of selectivity. Other options such as laser photolysis, in the case of thujone from *Salvia sp*. Extracts [32], or trapping on molecular imprinted polymers, in the case of safrole in nutmeg oil [33], have been reported or patented but their applicability at the industrial scale is not yet recognised.

#### 2.2.1. Tuning of Biological Properties

**Scheme 2:**
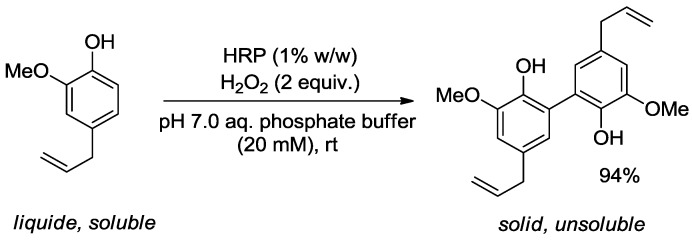
HRP-catalysed dimerisation of eugenol in the presence of H_2_O_2_.

In the following examples, we will focus on studies where only a group of molecules are transformed (*i.e*., alcohols) or even a single molecule (*i.e*., eugenol). It might be desirable to modify the biological properties of essential oils in case of beneficial or deleterious effect of one or more metabolites. In the context of toxic or allergen molecules, a model study on the removal of eugenol from rose essential oil by a bi-enzymatic strategy has been reported [34]. The principle relied on the use horseradish peroxidase (HRP) to oxidatively dimerise eugenol within the essential oil, turning this liquid soluble component into an unsoluble solid material, simply removed by filtration during the work-up (Scheme 2).

Although the enzymatic reaction was particularly efficient with pure eugenol, which was almost quantitatively converted to dieugenol, the reaction conditions could not be directly transferred to the transformation of essential oils. Indeed, to avoid side-reactions that could occur from the contact of H_2_O_2_ with unsaturated terpenes (limonene, pinenes …), a sequential procedure was developed, including the action of a second enzyme, catalase, to oxidise residual H_2_O_2_ to O_2_. Rose essential oil could therefore be submitted to this sequential procedure consisting in the activation of HRP by H_2_O_2_ during 1.5 h, followed by the addition of catalase (under H_2_O_2_-concentration monitoring by titration with KMnO_4_) and the final addition of the essential oil for 4 h. Chromatographic analysis (GC/MS) showed no remaining eugenol in the modified essential oil, and only slight side modifications of the concentration in citronellol and phenylethanol. The chemical analysis was followed by sensory analysis by triangular testing on modified and non-modified essential oils. A 12-people panel was used and the results showed no significant statistical difference between both samples at the 95% level of confidence. This work was the first example of the chemical modification of essential oils by enzymatic treatment and supported the relevance of using enzymes for the selective removal of toxic or hazardous compounds from essential oils without ruining their naturality.

#### 2.2.2. Tuning of Olfactory Properties

**Scheme 3:**
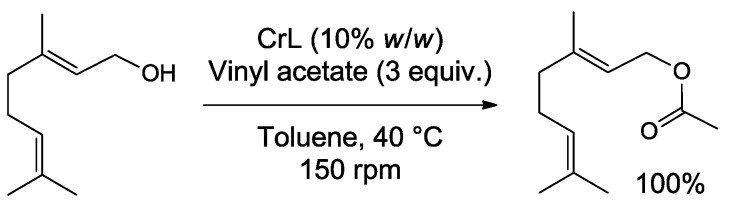
Example of acetylation of geraniol catalysed by *Candida rugosa* lipase (CrL).

The olfactory quality of an essential oil is correlated to the presence and concentration of impact odorant molecules. It is therefore possible to improve the olfactory quality of an essential oil by chemical modification, but such operation also result in the loss of the natural character of the product. Palmarosa (*Cymbopogon martinii*) essential oil, a cheap and abundant material with a rather limited interest as perfuming ingredient, could be modified by enzymatic treatment in a controlled manner resulting in improved olfactory properties [35]. Thanks to the large quantity of terpenic alcohols in palmarosa (including more than 80% of geraniol), the application of a lipase-based strategy allowed obtaining a variety of modified oils by combining various acyl donors and various rates of acylation, controlled by the equivalent of vinyl esters engaged as acyl donors. Interestingly, it was shown that the heavy odor of geraniol of the essential oil could be limited by its conversion to geranyl esters catalysed by lipase from *Candida rugosa*, bringing fruity notes of pear (Scheme 3). 

**Figure 3:**
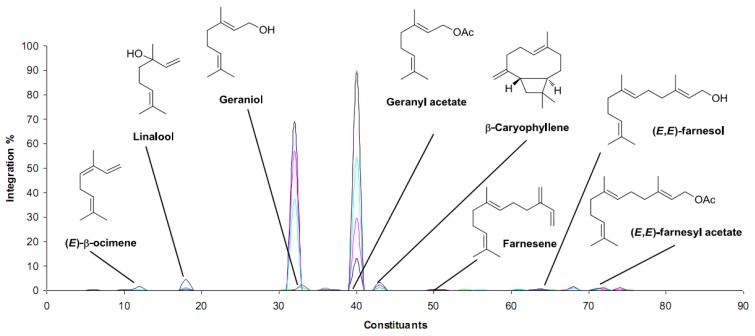
Comparison of modified and unmodified palmarosa essential oils upon CRL-catalysed acetylation based on GC-FID analysis (navy: original sample of palmarosa; pink: 20% acetylation; light blue: 40% acetylation; green: 80% acetylation; purple: 100% acetylation). See ref. 35 for analytical conditions and details.

Moreover, in this more balanced mixture, secondary compounds, masked in palmarosa essential oil, could also be perceived as undertones, as it is the case with spicy notes of cloves due to caryophyllene or the citrus/green notes of (*E*)-β-ocimene, linalool and farnesyl esters (Figure 3).

Since vinyl esters where primarily used as acyl donors, a second procedure was set up to save the naturality of the product using natural ethyl acetate both as the solvent and the acyl donor. Under these conditions, up to 100% acetylation could be observed applying prolonged reaction times (up to 96 h) and increased biocatalyst loading (to 60% *w*/*w*), in a reaction kinetically limited by a saturation phenomenon of the enzyme by EtOAc substrate. Alep rose essential oil could be similarly modified by lipases, resulting in modified olfactory properties [36]. The acylation of alcohols such as geraniol, citronellol and 2-phenylethanol, major constituents of rose essential oil, resulted in a significant shift of the olfactory properties of the product towards less rosy-floral notes.

**Figure 4:**
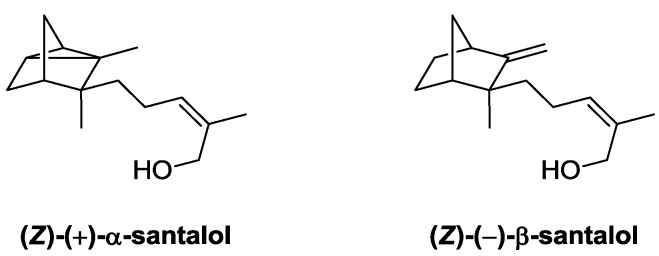
Isomers of santalol, the main impact odorants of sandalwood essential oil.

Sandalwood essential oil is a highly priced odorant material from different origins and for which more than 230 constituents have been identified [37]. In spite of this chemical diversity, it seems that the most important impact-odorant molecules are (*Z*)-(+)-α-santalol and (*Z*)-(–)-β-santalol, which are also the main constituents of the essential oil (Figure 4) [38].

**Figure 5:**
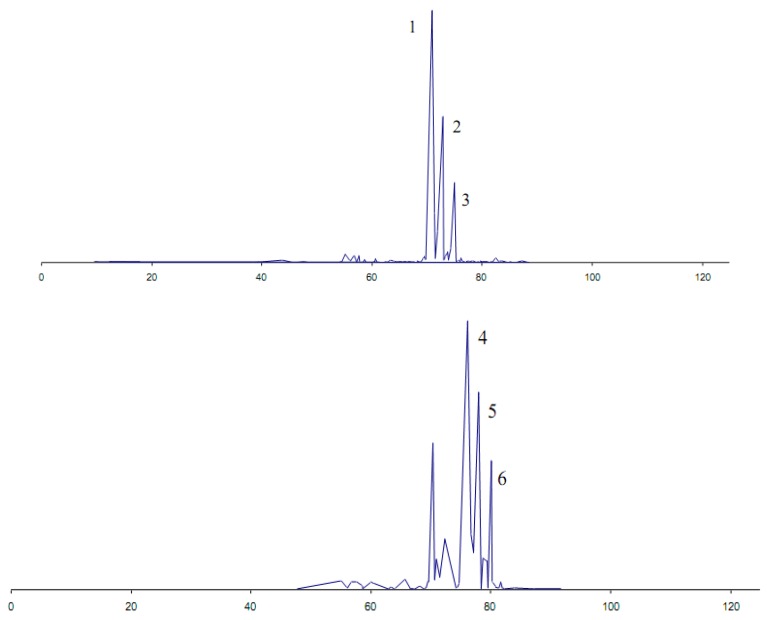
GC/MS chromatograms of a sandalwood essential oil from New-Caledonia before and after acetylation by enzymatic treatment. **1**: (*Z*)-(+)-α-santalol, **2**: (*Z*)-(–)-β-santalol, **3**: β-santalol isomer, **4**–**6**: acetylated forms of 1–3 (M = 262). Conditions: chromatograph Agilent 6890N coupled to an Agilent 5973 MS detector. Samples were analyzed on a fused-silica capillary column HP-1 (polydimethylsiloxane, 50 m × 0.20 mm i.d. × film thickness 0.33 μm). Carrier gas, helium, constant flow 1 ml/min, injector temperature, 250 °C, split ratio, 1:100, temperature program, 45 °C to 250 °C, at 2 °C/min then held isothermal (30 min) at 250 °C, ion source temperature, 230 °C; transfer line temperature, 250 °C; ionization energy, 70 eV; electron ionisation mass spectra were acquired over the mass range 35–400 amu.

The lipase-based strategy was applied to a sample of sandalwood oil New Caledonian (*Santalum austrocaledonicum*) [38]. As a result, the major alcohol constituents were acylated with acetyl, propionyl, or crotonyl groups in the presence of CrL (Figure 5). The modified essential oils lost their typical sandalwood odor, in favour of more common woody notes, together with immortelle, mushroom or blackcurrant undertones. The overall intensity of the olfactory properties was weaker in the modified sandalwood essential oils.

Enzymes belonging to the class of hydrolases naturally present in olives could be used in certain processing conditions to enhance the organoleptic quality of olive oil [39]. In the case of essential oils, obtained in most instances by distillation, such strategy could not viable. With citrus essential oils however, typically obtained by cold-pressing, this approach might be useful.

## 3. Conclusions

With a virtually infinite number of enzymes available from living organisms, including organisms resulting from directed-evolution, or with bioengineered biocatalysts, the repertoire of chemical reactions that could be catalysed by an enzyme is huge. Moreover, with the high selectivity and substrate specificity commonly achieved by enzymatic processes, the discovery a tailored treatment for the fine-tuning of essential oils properties, and of natural extracts in general, is likely to increase exponentially in a near future. In this review, we have seen some examples of optimisation of biological properties such as the bioactivity, the olfactory properties and the safety of a selection of essential oils (summarised in Table 1). Other properties could be modified by enzymatic treatment such as the physico-chemical properties: solubility in a given medium, lipophilicity, or even chromatographic elution of natural extracts components for analytical purposes.

**Table 1:** Summary of the uses of enzymes for the modification of essential oils presented in this review.

Enzymes	Essential oils/extracts	Effects	Properties modified	Ref.
Pre-treatment				
Cellulase, pectinase, protease, viscozyme	Cumin (*Cuminum cyminum L.*)	Yield improved	None	[18]
Cellulase, pectinase, protease, viscozyme	Celery (*Apium graveolens L.*)	Yield improved	None	[21]
Hydrolytic enzymes (extract)	Chili guajillo (*Capsicum annuum L.*)	Carotenoids/capsaicinoids recovery	Organoleptic	[19,20]
Cellulase, β-glucanase, pectinase, xylanase	Black pepper	Yield improved, caryophyllene content increased	Organoleptic	[22]
Cellulase, β-glucanase, pectinase, xylanase	Cardamom	Yield improved, α-terpenyl acetate content increased	Organoleptic	[22]
Cellulase, β-glucanase, pectinase, xylanase	Thymus (*Thymus capitatus*)	Yield improved, carvacrol content increased	Anti-microbial	[23]
Cellulase, β-glucanase, pectinase, xylanase	Rosemary (*Rosmarinus officinalis*)	Yield improved, 1,8-cineole content decreased	Anti-microbial	[23]
Extracts (β-glucosidase, α-rhamnosidase)	Bergamot (*Citrus bergamia* Risso)	Flavonoids content increased	Anti-oxidant	[24]
β-Glucosidase	Sweet olive (*Osmanthus fragrans* Lour)	Yield improved, nonanal, dihydro-β-ionol, and (*E*)-β-ionone contents increased	Organoleptic	[26]
β-Glucosidase	Arabian pea (*Psoralea bituminosa*)	Hex-3-en-1-ol and oct-1-en-3-ol contents increased	Organoleptic	[27]
β-Glucosidase and α-amylglucosidase	Clove (*Syzygium aromaticum* (L.) merr. and perry (Myrtaceae))	Eugenol, isoeugenol, farnesol, and nerolidol contents increased	Organoleptic	[28]
β-Glucosidase and pectinol C	Hyssop (*Hyssopus officinalis* L.)	Terpenyl alcohols, and phenolics contents increased	Organoleptic	[29]
**Post-treatment**				
Horseradish peroxidase	Rose (*Rosa* sp.)	Eugenol content decreased	Toxicity/allergenicity	[34]
*Candida rugosa* lipase	Palmarosa (*Cymbopogon martinii*)	Terpenyl alcohols contents decreased, terpenyl esters contents increased	Organoleptic	[35]
*Candida antartica* lipase B	Rose (*Rosa* sp.)	Terpenyl alcohols contents decreased, terpenyl esters contents increased	Organoleptic	[36]
*Candida rugosa* lipase	Sandalwood (*Santalum austrocaledonicum*)	α- and β-santol contents decreased in favor of their esters	Organoleptic	[38]
